# Cryoablation combined with allogenic natural killer cell immunotherapy improves the curative effect in patients with advanced hepatocellular cancer

**DOI:** 10.18632/oncotarget.17804

**Published:** 2017-05-11

**Authors:** Mao Lin, Shuzhen Liang, Xiaohua Wang, Yinqing Liang, Mingjie Zhang, Jibing Chen, Lizhi Niu, Kecheng Xu

**Affiliations:** ^1^ Department of Biological Treatment Center, Fuda Cancer Hospital, Jinan University School of Medicine, Guangzhou, China; ^2^ Fuda Cancer Institute, Guangzhou, China; ^3^ Hank Bioengineering Co., Ltd., Shenzhen, China; ^4^ Department of Oncology, Fuda Cancer Hospital, Jinan University School of Medicine, Guangzhou, China

**Keywords:** curative effect, percutaneous cryoablation, allogenic natural killer cell, hepatocellular cancer, progression-free survival

## Abstract

In this study, the clinical efficacy of cryosurgery combined with allogenic natural killer cell immunotherapy for advanced hepatocellular cancer was evaluated. From October 2015 to March 2017, we enrolled 61 patients who met the enrollment criteria and divided them into two groups: 1) the simple cryoablation group (Cryo group, *n* = 26); and 2) the cryoablation combined with allogenic natural killer cells group (Cryo-NK group, *n* = 35), the safety and short-term effects were evaluated firstly, then the median progression-free survival, response rate and disease control rate were assessed. All adverse events experienced by the patients were recorded, and included local (e.g., pain, pleural effusion, and ascites) and systemic (e.g., chills, fatigue, and fever) reactions, fever was more frequent. Other possible seriously side effects (e.g., blood or bone marrow changes) were not detected. Combining allogeneic natural killer cells with cryoablation had a synergistic effect, not only enhancing the immune function, improving the quality of life of the patients, but also reducing the expression of AFP and significantly exhibiting good clinical efficacy of the patients. After a median follow-up of 8.7 months (3.9 –15.1months), median progression-free survival was higher in Cryo-NK (9.1 months) than in Cryo (7.6 months, *P* = 0.0107), median progression-free survival who received multiple natural killer was higher than who just received single natural killer (9.7 months vs.8.4 months, *P* = 0.0011, respectively), the response rate in Cryo-NK (60.0%) was higher than in Cryo (46.1%, *P* < 0.05), the disease control rate in Cryo-NK (85.7%) was higher than in Cryo group (69.2%, *P* < 0.01). Percutaneous cryoablation combined with allogeneic natural killer cell immunotherapy significantly increased median progression-free survival of advanced hepatocellular cancer patients. Multiple allogeneic natural killer cells infusion was associated with better prognosis to advanced hepatocellular cancer.

## INTRODUCTION

Hepatocellular carcinoma (HCC), which is the most common type of primary liver cancer, due to usually discovered late and has a poor prognosis [1]. Although surgical resection is the best option to HCC, but many patients suffer bad factors such as cirrhosis, multiple tumors or other diseases, so they are unsuitable for surgical resection [2, 3]. Up to now, systemic chemotherapy dose not obviously increase survival advanced HCC patients [4, 5]. Radiation has toxicity to the normal liver, so it is limited method to treat HCC [6, 7]. Hence, the newer and more effective therapies for advanced HCC patients are needed.

Percutaneous cryoablation is a new method which can induce tumor necrosis, it is currently considered the best option for treating unresectable HCC [8]. Cryosurgery has many advantages which carried out successfully in many solid tumors (e.g., prostate cancer and RR) [9, 10], and also emerged as a new therapy pattern for HCC [11, 12].

There are some reports have stated that cancer occurence and development in HCC patients are affected by the tumor immune [13–18]. However, due to down-regulating the expression of MHC, tumor cells often immune escape [19] . Manipulating the immune system for therapeutic benefit in HCC patients has been studied for many decades [20–22]. NK cells are belonged to the innate immune system and play a importment role against cancer in the early time [23, 24]. With progress in the NK cell biology field and in understanding NK function, adoptive NK cell transfer has promising anti-tumor effects on various cancers [25–29], including liver cancer [30–34].

In our research, we prospectively investigated the clinical response of cryosurgery combining with NK cell immunology for advanced HCC, to elucidate a potential therapeutic strategy.

## RESULTS

### Identification of NK cells in the enrolled patients

**Figure 1:**
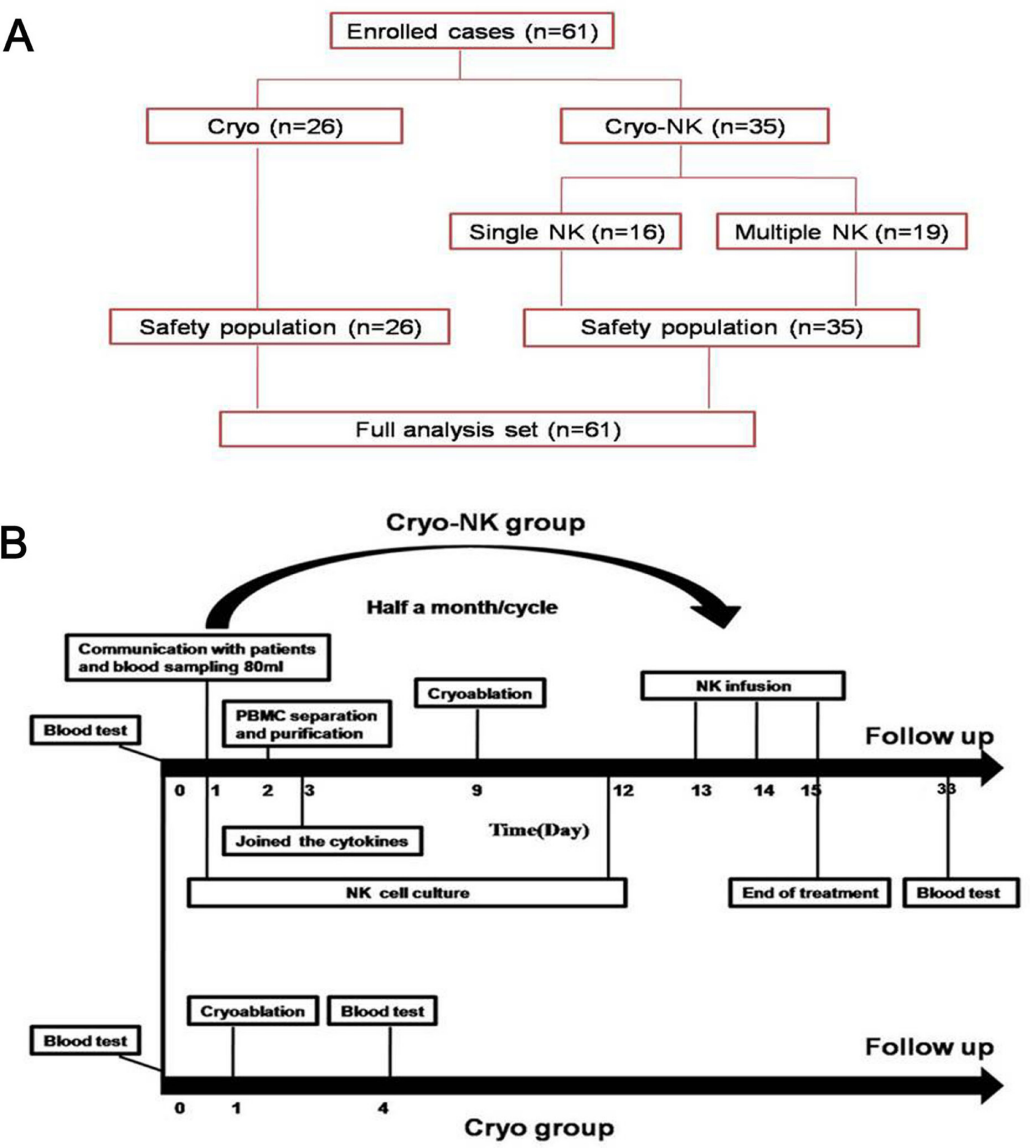
(**A**) The enrolled patients were allocated to cryoablation group (*n* = 26) and Cryo-NK group (*n* = 35, 16 patients underwent one course NK and 19 patients underwent ≥ 3 courses). (**B**) The treatment schedule. All of the enrolled patients’ kinsfolk were informed, and the peripheral blood was collected for isolation of NK cells seven days before cryoablation, cryoablation was carried out on Day 9 and Day 12, the cultured NK cells were intravenously infused on days 13 to 15, while only cryoablation was performed in the cryotherapy group.

**Figure 2:**
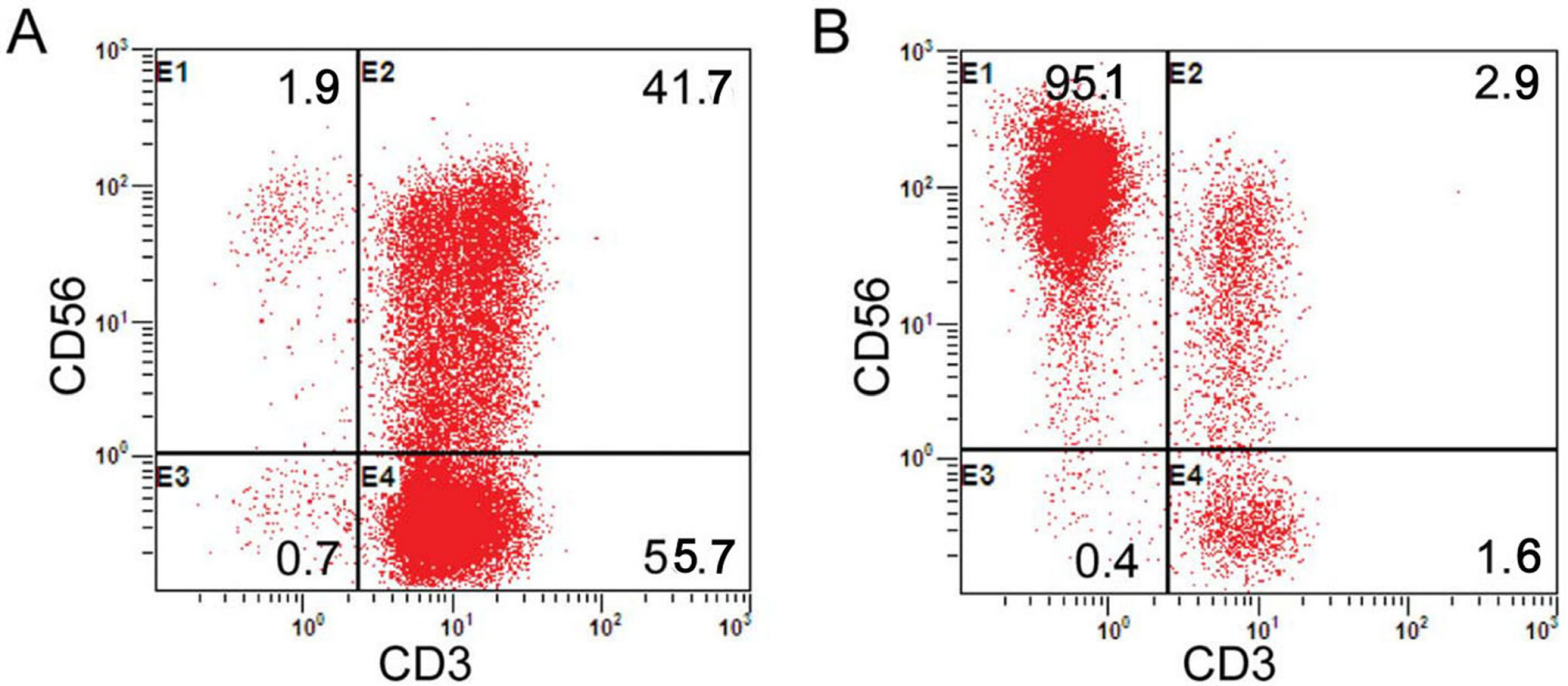
Proportion analysis of CD3-CD56+ cells (NK cells) (**A**) The proportion of NK cells before the expansion; (**B**) The proportion of NK cells after expansion on Day 9.

In line with the protocol described in Figure 1B, the peripheral blood of the enrolled patients’ kinsfolk was separated in the laboratory. Prior to the cell culture, the median fraction of the CD3^-^CD56^+^ population was 6.69% (range: 4.3% -14.9%). Following the cell culture and expansion, the viable cells exceed 90%. The median proportion of CD56+ cells was 86.1% (range: 71.6% - 96.1%). The median percentage, viable cells and the median proportion of CD56+CD3- cells were similar to our prior report [35]. The representative results are presented in Figure 2.

### Patient demographics

61 patients come around the world, China (*n =* 31), Indonesia (*n =* 11), Malaysia (*n =* 12) and Mid East (*n =* 7). The data were no statistical differences (Table 1, *P* > 0.05).

**Table 1:** Patient demographics

Patient characteristics before treatment	Cryoablation (*n* = 26)	Cryo-NK (*n* = 35)	*P* value
Gender (Male/Female)	14/12	18/17	*P =* 0.824
Median age (y)	56	61	*P =* 0.715
Child-Pugh Stratification			*P =* 0.763
Class A	11	16	
Class B	15	19	
Clinical stage (AJCC)			*P =* 0.864
III	12	17	
IV	14	18	
Karnofsky performance status			*P =* 0.744
70	13	14	
80	8	15	
90	5	6	
Hepatitis B/C+	12	14	*P =* 0.836
TACE	23	22	*P =* 0.922

AJCC, American Joint Committee on Cancer staging system; TACE, Transarterial chemoembolization.

### Safety and clinical efficacy evaluation

**Figure 3:**
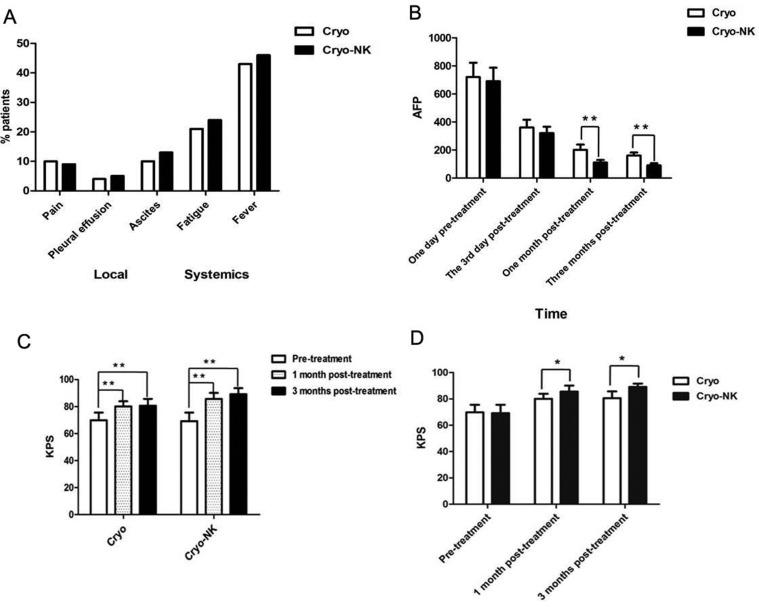
Safety and clinical efficacy evaluation (**A**)Among the patients, the most common reactions included fever, pain, pleural effusion, and ascites were nearly remitted compared with the patients oral at pre-treatment following symptomatic treatment; (**B**) There was no difference between the two groups at post-treatment day 3 (*P* > 0.05), but at 1 month and 3 months post-treatment, AFP expression was obviously lower in the cryo-NK group than in the cryo group; (**C**) Compared with the pre-treatment KPS, the scores of both groups were obviously improved post-treatment; (**D**) The KPS was higher in the cryo-NK group both one month and three months post-treatment. *P < 0.05; **P < 0.01.

All adverse events were recorded, including local (e.g., pain, pleural effusion, and ascites) and systemic (e.g., chills, fatigue, and fever) reactions, fever was more frequent. The adverse events was compared using a chi-square test; they were no differences (*P =* 0.8481; Figure 3A).

The pre-treatment immune test data of were merged and compared with post-treatment (Table 2). Aimed to the counts, all cells were exceed in the Cryo-NK group following treatment (*P* < 0.01), especially to NK, the number in Cryo-NK group was significantly higher (*P* < 0.001). Considering lymphocyte functionality, the level of Th1 cytokines was also improved in the cryo-NK group, the level of Th2 cytokine levels remained invariability.

**Table 2:** Comparison of the number and function of lymphocytes

Test items of lymphocyte	Test results		
	Pre-treatment (*n*=61)	Cryoablation (*n*=26)	Cryo-NK (*n*=35)
Number (cell/μL):			
Total T cell	1456 ± 52	1623 ± 67*	1812 ± 91**
CD8+T cell	621 ± 7	727 ± 29*	754 ± 11**
CD4+T cell	735 ± 30	821 ± 35*	856 ± 33**
NK cell	372 ± 31	552 ± 63*	786 ± 73***
B cell	309 ± 11	465 ± 33**	551 ± 41*
Function (pg/mL):			
IL-2	9.9 ± 3.4	17.6 ± 3.5**	23 ± 4.6***
TNF-β	3.8 ± 2.2	9.8 ± 2.5***	14 ± 2.5***
IFN-γ	4.8 ± 3.3	10.8 ± 3.6**	16 ± 37***
IL-4	10.2 ± 2.1	10.1 ± 3.4	11.2 ± 3.6
IL-6	13.2 ± 3.9	15.2 ± 4.5	15.5 ± 7.9**
IL-10	9.2 ± 2.7	9.3 ± 2.3	10.1 ± 3.4

Note: All cell subsets and cytokines were analyzed via a Dunnett’s multiple comparison test (one-way ANOVA). NK cell, natural killer cell; IL, interleukin; TNF, tumor necrosis factor; IFN, interferon *P < 0.05; **P < 0.01; ***P < 0.001.

AFP expression was higher than normal at 1 day pre-treatment and decreased gradually at day 3, and 1 month and 3 months post-treatment in the two groups (Figure 3B). There was no difference between the two groups at post-treatment day 3 (*P* > 0.05), but at 1 month and 3 months post-treatment, AFP expression was obviously lower in the Cryo-NK (Figure 3B, *P* < 0.01), even there were 13patients within normal range who received Cryo-NK therapy.

The pre-treatment KPS of the Cryo- and Cryo-NK groups was 68.7 ± 6.5 and 70.1 ± 3.7, respectively, 78.6 ± 4.2 and 82.9 ± 5.1 one month post-treatment, respectively, and 81.2 ± 4.7 and 90.1 ± 1.6 at three months post-treatment, respectively. Compared with the pre-treatment KPS, the scores of both groups were obviously improved post-treatment (Figure 3C; *P <* 0.01); however, the KPS was higher in the Cryo-NK group both one month and three months post-treatment (Figure 3D; *P* < 0.05).

**Figure 4:**
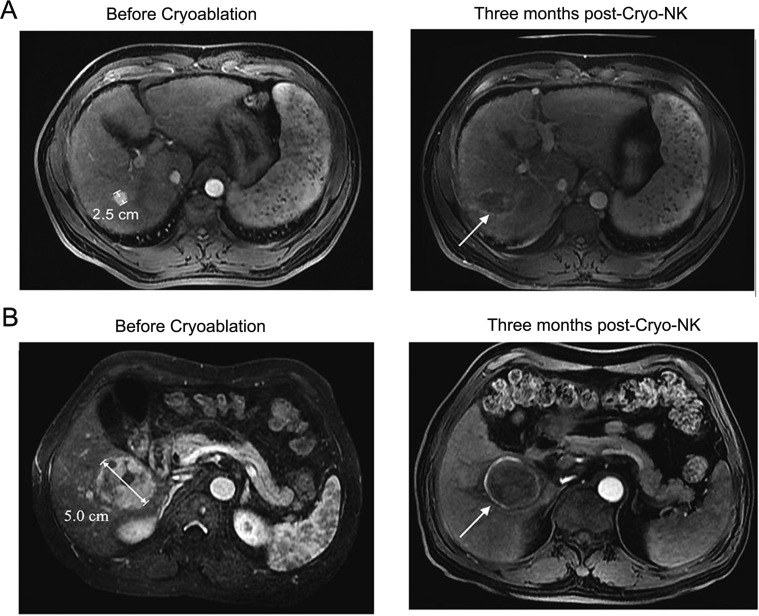
MRI images of 2 representative cases achieved CR at three months post-Cryo-NK (**A**) Case #1, a 50-year-old male, stage III, a maximum HCC nodule of 2.5 cm, MRI showed no enhancement in the occupying lesion, with mild shrinkage of the area; (**B**) Case #2, a 48-year-old female, stage IV, a maximum HCC nodule of 5.0 cm, MRI showed a lesion with a large area of necrosis.

The clinical efficacy between the two groups was observed three months post-treatment. The maximum transverse diameters are listed in Table 3. The tumor volume was obviously decreased post-treatment in both two groups; however, three months post-treatment, the maximum tumor diameter in cryo-NK was smaller than Cryo (*P* < 0.01). In Table 4, CT value was obviously decreased post-treatment, the CT value three months post-treatment in the Cryo-NK group was lower than that of the Cryo group (*P* < 0.05). During the follow-up period, there was no patient died; the number of CR in Cryo was 5 but 9 in cryo-NK (Table 5). In addition, the RR in cryo-NK was 60.0% higher than 46.1% in Cryo (*P* < 0.01), similarly, DCR in cryo-NK was obviously higher than patients who just received Cryo (85.2% vs. 69.5%, *P* < 0.01). The representative results are shown in Figure 4.

**Table 3:** The maximum transverse diameter of the lesions pre- and post-treatment

Time	Maximum diameter (mm)		*P* value
	Cryoablation	Cryo-NK	
Pre-treatment	4.68 ± 3.13	4.59 ± 3.38	*P* > 0.05
1 month post-treatment	4.12 ± 3.00	4.07 ± 3.24	*P* > 0.05
3 months post-treatment	4.04 ± 3.01	3.68 ± 2.46	*P* < 0.05

**Table 4:** The CT value of lesions pre- and post- treatment

Time	Plain CT value (Hu)		*P* value
	Cryoablation	Cryo-NK	
Pre-treatment	43.01 ± 7.66	42.29 ± 7.12	*P* > 0.05
1 month post-treatment	25.58 ± 2.06	24.87 ± 3.12	*P* > 0.05
3 months post-treatment	24.87 ± 3.25	23.21 ± 3.68	*P* < 0.05

**Table 5:** Clinical response between the two groups at three months post-treatment

	Total	Cryo	Cryo-NK	*P* value
Number	61	26	35	*P* > 0.05
CR	13	5	9	*P* > 0.05
PR	19	7	12	*P* < 0.05
SD	15	6	9	*P* > 0.05
PD	14	8	5	*P* > 0.05
RR (%)	52.4	46.1	60.0	*P* < 0.05
DCR (%)	77.1	69.2	85.7	*P* < 0.01

Clinical responses were evaluated according to Response Evaluation Criteria in Solid Tumors version 1.1. CR, complete response; PR, partial response; SD, stable disease; PD, progressive disease; RR, response rate; DCR, disease control rate.

**Figure 5:**
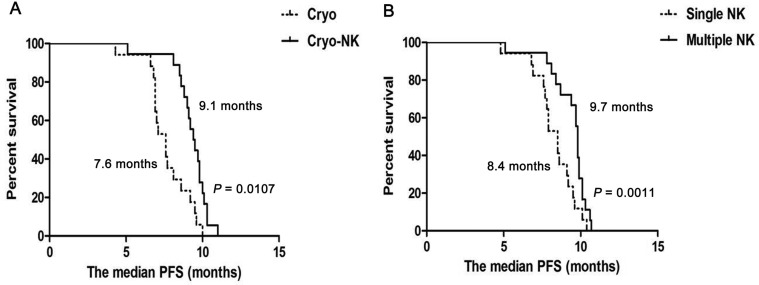
The median PFS from cryoablation (**A**) The median PFS was higher in Cryo-NK (9.1 months) than in Cryo group (7.6 months, *P* = 0.0107); (**B**) The median PFS who received multiple NK was higher than who just received single NK (9.7 months vs.8.4 months, *P* = 0.0011, respectively).

After a median follow-up of 8.7 months (3.1 –14.3months), median PFS was higher in Cryo-NK (9.1 months) than in Cryo group (7.6 months, *P* = 0.0107, Figure 5A), median PFS who received multiple NK was higher than who just received single NK (9.6 vs.8.4 months, *P* = 0.0011, respectively, Figure 5B).

## DISCUSSION

For the majority of patients with advanced HCC, tumor is not suitable to resect. With many advances and imaging, cryoablation becomes successfully for treating HCC [36–38]. Ar-He cryoablation is a novel micro-invasive treatment of tumor, which tumors are frozen and then left *in situ* to be reabsorbed. Cryoablation is a development technology with minimally invasive treatment of tumor in recent years, can quickly damage to tumor tissue, reduce tumor load. In this study, cryoablation was showed a safety and efficacy to advanced HCC patients for short term observation. However, due to the vast majority of advanced HCC patients have been confirmed the existence of the distal metastasis, cryoablation only treat primary tumors or metastases, possible role limited for advanced HCC cell metastasis.

It is increasingly clear that cancer occurence and development in HCC patients are affected by tumor immune [39, 40]. The recent success of immunotherapy has highlighted the potential of immune-based therapy approaches for advanced HCC treatment [21, 41, 42]. Our previous research of Cryo-DC/CIK treatment with in metastatic pancreatic cancer [43] and HCC [44] has displayed a good clinical outcome. After cryoablation, account of continued antigen released, which could stimulate the immune system sequentially, but CIK could not kill the low tumor burden directly, so we brought in NK cells technology by understanding NK function. In our study, we attempted to investigate the safety and clinical efficacy of percutaneous cryoablation combined with allogeneic natural killer cell therapy for treating advanced HCC, then the median PFS, RR and DCR were assessed. To our surprise was that allogeneic NK cells combined with cryosurgical for advanced HCC exhibited a synergistic effect, significantly enhanced the immune function of patients (Table 2). The increasing number of NK cells after allogeneic NK cells immunotherapy may be related to ectogenic NK cells amplified, because NK cells were infused in the period of logarithmic phase which owned the best activity and amplified sequentially. Moreover, combined treatment can markedly improve the median PFS prolonger, and the median PFS was higher in patients who received multiple NK than who just underwent only one course NK, also to RR and DCR (both *P* < 0.05). Facts proved that this comprehensive therapy was also safety and efficacy.

For decades, natural killer (NK) NK cells existed as “non-specific” killer cells were different from CTL or other immunocytes identified the target. We have learned that NK cells are trained to recognize “non-self” histocompatibility antigens (human leukocyte antigen, HLA) on the surface of cells through their killer cell immunoglobulin-like (KIR) receptors. Recent discoveries that better explain how NK cells recognize and kill their targets and their ability to produce immune-active cytokines have made them more attractive tools for immunotherapy. In view of this, we brought NK cells into this clinical research. In our previous report [45], showed a good outcome for allogenic NK cell immunotherapy to advanced renal cell cancer and metastasis pancreatic cancer [35]. Therefore, we used allogeneic NK cell adoptive therapy in this study. Facts proved that it was tolerant and efficacy. But whether the current number and purity of NK cells would be the optimal dose or not which is worthy us considering, maybe, we will carry out the dose grope in the future.

In conclusion, in this single-center prospective study, we provided evidence that allogeneic NK cell therapy combined with percutaneous cryoablation exhibited a favorable outcome for advanced HCC patients, indicating a potential novel therapeutic strategy.

## MATERIALS AND METHODS

### Ethics

This study was approved by the Ethics Committee of Guangzhou Fuda Cancer Hospital. In accordance with the Declaration of Helsinki, written informed consent was obtained from each participant at the Fuda Cancer Hospital.

### Patients

This was a prospective study of the therapeutic effects of a combined treatment strategy on HCC patients enrolled from October 2015 to March 2017. We enrolled 61 patients using the following criteria: 1) expected survival > three months; 2) aged between 20 to 80 years; 3) Karnofsky performance status > 70; 4) the following parameters were normal: platelets ≥ 80 × 10^9^/L; white blood cells ≥ 3 × 10^9^/L; neutrophils ≥ 2 × 10^9^/L; hemoglobin ≥ 90 g/L; prothrombin time international normalized ratio, 0.8–1.5; adequate hepatic function (bilirubin < 20 μM, aminotransferase < 60 U/L) and renal function (serum creatinine < 130 μM, serum urea < 10 mM), 5) all patients had confirmed HCC by pathology; 6) the absence of level 3 hypertension, severe coronary disease, myelosuppression, respiratory disease, acute or chronic infection, and autoimmune diseases. The enrolled patients were allocated to Cryoablation group (*n* = 26) and Cryo-NK group (*n* = 35, 16 patients underwent one course NK and 19 patients underwent ≥ 3 courses, Figure 1A) .

### Percutaneous cryoablation

The percutaneous puncture point was determined by computed tomography (CT). All cryoablations were performed with the commercially available Cryocare Surgical System (Endocare, Irvine, CA, USA) using argon and helium gas as the cryogen. Cryoprobes (3, 5 or 8 mm) were inserted into the center of the tumor mass under CT guidance and two freeze/thaw cycles were performed, each reaching a temperature between −125°C and −150°C for 15 min followed by rewarming at 15–20°C. A margin of at least 1 cm of normal breast tissue was frozen circumferentially around the tumor. For masses larger than 5 cm in diameter, two or three cryoprobes were placed within the center and periphery of the tumor, to ensure freezing of the entire mass. After cryoablation, antibiotics and haemostatic agents were administered and vital signs such as blood pressure, pulse, respiration and blood oxygen saturation were monitored routinely.

### CT and MRI examination

HCC patients confirmed by pathology were required to undergo dynamic CT or MRI one week pre-treatment, with a follow-up at one and three months post-treatment. The maximum diameter and CT value were measured and compared between the pre- and post- treatment values. A comprehensive analysis combined with the clinical data was completed together by two experienced nuclear medicine physicians who had multiple years of experience in making CT diagnoses.

### NK cell therapy

Clinical-grade NK cells were cultured using clinical-grade reagents and under good manufacturing practice conditions. The human high activity NK cell *in vitro* preparation kit was used (Hank Bioengineering Co. Ltd, Shenzhen, China) that contained chimeric active cellular factors on K562 cell membranes [46], plasma treatment fluid, lymphocyte culture fluid additives, serum-free medium additives and cell infusion additives. This kit is intended for expanding and activating NK cells in peripheral blood mononuclear cells *in vitro* to prepare NK cells of higher quantity, purity and activity, namely highly activated NK (HANK) cells [47]. Blood samples from the patient and donors were analyzed using the TIANamp Blood DNA kit (Tiangen Biotech Co., Ltd., Beijing, China) and KIR/HLA-C allotypes Genotyping Low Resolution kit (Tianjin Super Biotechnology Developing Co., Ltd., Tianjin, China). Approximately 8–10 billion HANK cells may be harvested after culture from 80 ml of peripheral blood using NK cell serumfree medium and culture bags (Tianjin Haoyang Biological Manufacture Co., Ltd, Tianjin, China). Cell counting and quality control inspection are commonly performed on day 12 of culture, and the quality indicators include ≥8 billion total cells with ≥ 90% living cells, ≥ 85% CD3−/CD56+ cells [47], ≤ 1 EU/ml endotoxin, ≥ 80% cell killing activity against K562 target cells [47], and bacteria-, fungi- and mycoplasma-negative culture. After 12 days of cell culture, the NK cells were divided into three groups and intravenously infused into the patients from Day 13 to 15. Each patient must two cycles NK therapy continuously as one course.

NK cells were generated according to previously published protocols [47], 80 ml peripheral blood from allogenic donors was drawn 7 days before cryoablation and the immunotherapy was given 3days after cryoablation.

For donor selection, the killer cell immunoglobulin-like receptors (KIRs) genotyping should be mismatched to the human leukocyte antigen (HLA) class I molecules of the patient [47–51]. We used PCR-SSP to detect the KIR/HLA-Cw which can get the result on the day.

### Cryo-NK therapy procedure

All of the enrolled patients’ kinsfolk were informed, and the peripheral blood was collected for NK cell isolation seven days before cryoablation. Cryoablation was carried out on Day 9 and Day 12, and the cultured NK cells were infused intravenously from Days 13 to 15 (Figure 1B).

### Safety and curative effect evaluation index

#### Adverse events

The most common adverse reactions were recorded and included local (e.g., pain, pleural effusion, and ascites) and systemic (e.g., chills, fatigue, and fever) reactions.

#### Detection of immune function

2 ml peripheral blood from patients was drawn 1 day before cryoablation and 3 days after cryoablation or one course NK cells therapy (Figure 1B). BD Multitest 6-color TBNK Reagent (BD Biosciences, USA) was used to detect the number of CD3+CD4+ cells (95% range: 441–2156 cells/μl), CD3+CD8+ cells (95% range: 125–1312 cells/μl), total CD3+ cells (95% range: 603–2990 cells/μl), CD3-CD19+ cells (95% range: 107–698 cells/μl) and CD3-CD16+CD56+ cells (95% range: 95–640 cells/μl). BD Cytometric Bead Array (CBA) Human Th1/Th2 Cytokine Kit II (BD Biosciences, USA) was used to detect the expression levels of IL-2 (95% range: 8–12.5 pg/ml), IL-4 (95% range: 3.5–6 pg/ml), IL-6 (95% range: 2.7–8.5 pg/ml), IL-10 (95% range: 1.8–4 pg/ml), tumor necrosis factor (TNF; 95% range: 1.7–2.5 pg/ml) and IFN-γ (95% range: 1.5–4 pg/ml). The number and function of lymphocytes in the peripheral blood of patients were tested according to the protocols given in the instruction manuals. Results above or within the reference range were considered to indicate normal immune function. When one or more values were below the reference range, it was considered to indicate immune dysfunction.

#### Quality of life (QOL)

By karnofsky score standard as an index, after treatment, KPS increased ≥ 10 divided into QOL improved, increased < 10 divided into QOL stable, KPS reduced ≥ 10 divided into QOL lower.

### AFP

Using electrochemical luminescence immunity analyzer and accessory kit (Roche Cobase 411) to detect serum AFP concentration at 1 day pre-treatment and at day 3, 1 month, 3 months post-treatment, carried out in accordance with the instruments and reagents. AFP normal range: 0–20 ng/ml.

#### Imaging change

The WHO first published the evaluation standards for the tumor curative effect for the main study objective of observing tumor changes [52]. According to the degree of change in the largest transverse diameter, the therapeutic effect is divided into a complete response (CR), partial response (PR), stable disease (SD), and progressive disease (PD). To accurately observe the therapeutic effects, the total area of all tumors before and after treatment was compared. The recent curative effect must be maintained for more than four weeks, and CR+PR represented the effective rate (RR).

#### Follow-up

The patients were required to undergo plain CT and enhanced CT at 1 week pre-treatment, and followed at 1 month and 2 months post-treatment. Imaging procedures were conducted every 3 months thereafter. The endpoints of interest were progression-free survival (PFS). PFS was defined as the interval between Cryo and local relapse, distant metastasis, or death, whichever occurred first.

#### Evaluation and statistical analysis

Complications were recorded and classified in accordance with the Common Terminology Criteria of Adverse Events v4.0. Radiographic local tumor control was assessed using image-guided tumor ablation criteria [53]. The basic characteristics of the two groups were compared using the chi-square test; immunity detection result data are presented as the mean ± standard deviation; the changes of imaging were compared using the Student’s *t*-test; local and systemic adverse events were marked in the nursing records and compared using the Chi-square test. Significant differences were indicated by *P* < 0.05, *P* < 0.01 or *P* < 0.001. All analyses were conducted using GraphPad software (GraphPad, San Diego, CA, USA).

## Abbreviations

Hepatocellular cancer: HCC
natural killer: NK
progression-free survival: PFS
response rate: RR
disease control rate: DCR
major histo-compatibility complex: MHC
killer cell immunoglobulin-like receptors: KIRs
